# Retrospective perceptual distortion of position representation does not lead to delayed localization

**DOI:** 10.2478/v10053-008-0128-7

**Published:** 2013-03-15

**Authors:** Ricky K. C. Au, Fuminori Ono, Katsumi Watanabe

**Affiliations:** 1 Research Center for Advanced Science and Technology, The University of Tokyo, Japan; 2 Japan Society for the Promotion of Science, Tokyo, Japan; 3 Faculty of Education, Yamaguchi University, Japan

**Keywords:** attention, distortion, reaction time, retrospective, space

## Abstract

Previous studies have reported retrospective influences of visual events that occur after target events. In the attentional attraction effect, a position cue presented after a target stimulus distorts the target’s position towards that of the cue. The present study explored the temporal relationship between stimulus presentation and reaction time (RT) in this effect in two experiments. Participants performed a speeded localization task on two vertical lines, the positions of which were to be distorted by an additional attentional cue. No significant difference in RTs was found between the conditions with simultaneous and delayed cues. RTRT was modulated by the perceived (rather than physical) alignment of the lines. In Experiment 2, we manipulated the strength of attentional capture by modulating the color relevance of the cue to the target. Trials with cues producing stronger attentional capture (with cues of a different color from the targets) were found to induce apparently stronger distortion effects. This result favors the notion that the observed repulsion and attraction effects are driven by attentional mechanisms. Overall, the results imply that the attentional shift induced by the cue might occur rapidly and complete before the establishment of conscious location representation of the cue and the target without affecting overall response time.

## Introduction

In every moment, the sensory system constantly collects information from the physical world in order to construct representations of the perceptual reality. Before incoming information can be transformed into a conscious perceptual experience, it is processed through many unconscious processes for a certain amount of time. One central question is whether the physical flow of sensory events always determines the temporal properties of perceptual experiences and behavioral re-actions. As described by Dennett and Kinsbourne (1992) with a critical stance, in the Cartesian Theater view, every detail relevant to the sensory event must go through processes of integration and interpretation in order to generate a single, “final” percept. Consequently, this view would predict that a delay in stimulus presentation should lead to a comparable amount of delay in conscious perception and response; therefore, the observer should be able to react to what reaches consciousness first at an earlier moment.

However, our conscious perception does not always reflect physical events. Various visual masking and priming paradigms have been applied to render visual stimuli invisible in order to study the influence of unconscious processing on conscious perception (e.g., Blanco & Soto, 2009; Breitmeyer, Ogmen, & Chen, 2004; Chou & Yeh, 2011; Mulckhuyse, Talsma, & Theeuwes, 2007; Ogmen, Breitmeyer, & Melvin, 2003). In addition, conscious perception can also be influenced by events that occur after the target event. For example, in experiments investigating the flash-lag effect, it was found that the conscious percept attributed to the instance of a flash depended on events that occurred 80 ms afterwards (Eagleman & Sejnowski, 2000). The line-motion illusion, which is generated by local attentional capture (Hikosaka, Miyauchi, & Shimojo, 1993), can also be altered by retrospective influence (Eagleman & Sejnowski, 2003). In exploring the influence of attention on visual position, Suzuki and Cavanagh (1997) reported the attentional repulsion effect, in which a visual cue presented before a target stimulus that drew spatial attention altered the perceived position of a target stimulus, such that the target appeared to be repelled away from the location where the cue had been presented. In contrast to this prospective effect, Ono and Watanabe (2011) found that the cue can also shift the perceived position of the target when the cue is presented after the target, but in an opposite direction from that observed under the attentional repulsion effect; this tendency was named the *attentional attraction effect*. Successive presentation of stimuli can also cause mislocalization of a target stimulus relative to a comparison stimulus. Bocianski, Müsseler, and Erlhagen (2008) reported that the direction of mislocalization reversed as the stimulus onset asynchrony (SOA) between the target and the comparison stimulus increased; further, the extent of mislocalization increased as the spatial distance between the stimuli decreased. Continuing in the same line of research, Bocianski, Müsseler, and Erlhagen (2010) later discovered that attention could modulate the mislocalization effect: Distributed attention increased mislocalization, but focused attention eliminated it. This implied that our subjective perception of spatial locations depends on how and to where in the visual field we orient our attention. All these phenomena demonstrate that our conscious representations might be continuously updated to reflect the temporal dynamics of the considered events.

In the original demonstration of the attentional repulsion effect, Suzuki and Cavanagh (1997) proposed that the perceived position of visual objects is represented by a centroid of distribution of position-coding units. The sudden presentation of peripheral cues captures the observer’s attention and shifts the centroid to the opposite of its preexisting direction, resulting in mislocalization of the target’s position towards that direction. However, such explanation is not compatible with the attentional attraction effect, which shows that the effect of attentional capture can be reversed by presenting the cue after the target. Besides the position-coding account, it has been proposed that the shift of visual attention between cue and target stimuli may be responsible for the observed attentional repulsion and attraction effects (Chien, Ono, & Watanabe, 2011; Ono & Watanabe, 2011). According to the two-process assumption regarding the processing of visual stimuli mentioned by Müsseler and Aschersleben (1998), the presentation of a visual stimulus triggers both a coding process and an attentional process, which take place simultaneously. On the basis of this assumption, Müsseler and Aschersleben proposed that what an observer subjectively perceives is not the state when the attentional shift is initiated, but the state when it is completed. In agreement with this idea, previous studies suggested that the attentional repulsion effect might be caused by overshooting of an attentional shift from the cue to the target (Shim & Cavanagh, 2004; Yamada, Kawabe, & Miura, 2008): The cue first captures attention, and the subsequent presentation of the target recaptures attention in a way such that the shift causes the perceived position of the target to overshoot its veridical position, resulting in an apparent repulsion effect. On the basis of this notion, Ono and Watanabe (2011) proposed that the attraction effect occurs according to a similar principle, namely, that the dynamic attentional shift from the target to the cue in positive SOA conditions shifts the perceived location of the target towards the cue. The observer would only be conscious of the settled state configuration as the final perception, after the unconscious process of attentional shift totally completed (Müsseler & Aschersleben, 1998).

The objective of the present study was to investigate the relationship between the temporal characteristics of stimulus presentation (i.e., cue-target SOA) and behavioral responses (reaction time; RT) in target localization tasks that generate the attentional repulsion and attraction effects. We were particularly interested in localization RT in the attentional attraction effect, as it concerns the retrospective effect on a prior target stimulus. If (a) the processing rates of the target and cue are comparable, (b) the conscious perception faithfully reflects the processing order (in the manner predicted by the Cartesian Theater view), and (c) the speed of behavioral reaction reflects the flow of conscious perception, then the conscious perception of distorted target position should be established with a delay that reflects the cue-target SOA; hence, the localization RT should also include a comparable delay compared to that under simultaneous presentation of cue and target. However, if the RT observed with positive cue-target SOA (i.e., condition favoring the attentional attraction effect) does not differ from that under the simultaneous presentation condition, it would suggest that the relative time that the observer perceives the events (perceptual experience) does not reflect the actual internal processing times of these events. This would further imply that the attentional shift takes place at an unconscious level and that the observer would only be aware of the final version of the settled configuration (Müsseler & Aschersleben, 1998; Ono & Watanabe, 2011). For the negative cue-target SOA condition (i.e., the condition favoring the attentional repulsion effect), a comparison of RT against that in the no-cue control condition might indicate whether additional processing of the cue (and its influence on the perceived position of the target) might delay the overall speed of the cognitive processing involved.

## Experiment 1: Reaction time and the attentional repulsion/attraction effects induced under varied stimulus onset asynchronies

### Methods

#### Observers

Six volunteers as well as two of the authors (R.A. and O.F.) participated in the experiment. Except for R.A. and O.F., all observers were naďve as to the purpose of the study. All had normal or corrected-to-normal vision, and their informed consent was obtained prior to the experiment.

#### Apparatus and stimuli

The visual stimuli were created in MATLAB 7.3.0 (MathWorks, USA) using the Psychophysics Toolbox extensions (PsychToolbox 3.0.8; Brainard, 1997; Pelli, 1997) and were viewed on a CRT monitor with a refresh rate of 100 Hz (resolution = 1280 × 960 pixels); presentation was controlled by a personal computer running the Windows XP operating system. Observers were seated at a distance of 60 cm from the monitor screen in a dark and quiet room. A chin rest was used to fix the viewing distance of the observer.

White stimuli (luminance = 14.02 cd/m^2^) were presented against a black background (luminance = 0.022 cd/m^2^). A small, circular disc (diameter = 0.113°) positioned at the center of the screen was presented as a fixation point. The two types of cue stimuli consisted of two circles (diameter = 0.567°) placed diagonally either in top-left and bottom-right (L cue) or top-right and bottom-left (R cue) fashion. Each circle was displaced from the center of the screen both horizontally and vertically by 2.267°. The targets were two vertical bars (length = 0.567°, width = 0.0283°) presented 1.7° above and below the center of the screen. The upper bar was randomly presented at 21 different positions (with 0°, ±0.0283°, ±0.0567°, ±0.0850°, ±0.113°, ±0.142°, ±0.170°, ±0.198°, ±0.227°, ±0.255°, or ±0.283° horizontal displacement; i.e., 0 ±10 pixels) above the fixation point, and the lower bar was shown at 21 positions with the same horizontal displacements from the screen center but in the opposite direction from the upper bar. Before the start of the experiment, the observers were explicitly asked to remain fixated on the fixation point throughout the experiment; since the target bars were presented at random positions in each trial, the observers did not know in advance at which positions the target bars would appear. The role of eye movements in the experiment is thus assumed to be negligible.

#### Experimental design and procedure

**Figure 1.**
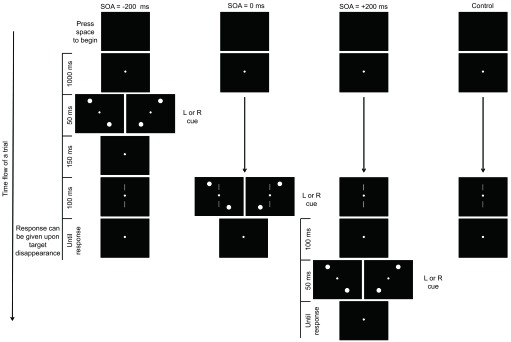
Flow of an experimental trial for each of the four conditions in Experiment 1. L cue stimuli consisted of circles placed diagonally in top-left and bottom-right fashion. R cue stimuli consisted of circles placed diagonally in top-right and bottom-left fashion. SOA = the stimulus onset asynchrony.

In each trial, the observer first saw a blank screen and pressed the space bar on the keyboard to initiate the trial (see Figure 1). The fixation dot then appeared and remained on the screen until the end of the trial. The target stimulus (100 ms) was always displayed 1,200 ms after the space bar had been pressed. The cue stimulus (50 ms) was presented with various cue-target SOAs: 200 ms before target onset (in the -200 ms SOA condition), simultaneously with target onset (in the 0 ms SOA condition), or 200 ms after target onset (in the +200 ms SOA condition). The experiment also included a control condition in which no cue was presented.

The observer was instructed to judge whether the position of the upper bar appeared to be to the left or right of that of the bottom bar and to press either the left or the right button of the computer mouse, respectively, as quickly as possible after the target disappeared. RT data were collected for each trial.

Each block was composed of 336 trials (4 SOA conditions × 2 cueing conditions × 21 target positions × 2 repetitions, in pseudorandom order) and took about 20 min to complete. The experiment included 15 blocks (total number of trials = 336 trials/block × 15 blocks = 5,040 trials) with breaks of at least 3 min between each block.

### Results

**Figure 2.**
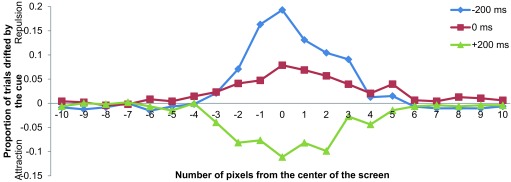
Magnitudes of attentional repulsion and attraction effects plotted against target positions in Experiment 1.

The magnitudes of the attentional repulsion and attraction effects were calculated as the difference between the proportions of “right” responses among the L cue and R cue trials in a given SOA and target position condition, divided by 2 (also refer to Ono & Watanabe, 2011, for this calculation). Positive values indicated repulsion, and negative values indicated attraction. In order to investigate the judgment responses and RTs in the +200 ms condition properly (in that condition, we wanted to make sure the observer had seen the cue before giving a response) and assuming that the attentional effects occur promptly and vanish within a short period (e.g., 1,000 ms), the data from trials with RTs shorter than 200 ms or longer than 1,000 ms were excluded from analysis (the average percentages of trials discarded from each of the conditions were as follows: -200 ms = 1.37%; 0 ms = 0.79%; +200 ms = 0.95%; control = 0.63%; overall = 0.94%). Figure 2 summarizes the average effects at each of the 21 target locations and three SOA conditions. The control condition was not included because no cue was presented in that condition, and hence the effects could not be calculated.

In line with previous results (Ono & Watanabe, 2011), the repulsion effect was observed when the positional cue was presented before the target (SOA = -200 ms), and the attraction effect was observed when the cue was presented after the target (SOA = +200 ms, cf. Figure 2). A 3 × 21 (SOA × Target Position) repeated-measures ANOVA on the proportion of “right” responses revealed significant main effects of SOA, *F*(2, 14) = 13.035, *p* < .001, and Target Position, *F*(20, 140) = 2.965, *p* < .001, and a significant SOA × Target Position interaction, *F*(40, 280) = 8.076, *p* < .001. Both the repulsion and attraction effects peaked at positions near the center of the screen where the vertical bars were physically close to each other and therefore their relative positions were less clear to the observers. Post-hoc pairwise comparisons (adjusted for multiple comparisons) using Ryan’s (1960) method were conducted to examine the differences in the mean magnitudes of the effects among the three SOA conditions. Significant differences were found between the -200 ms and +200 ms conditions and between the 0 ms and +200 ms conditions (both at *p* < .05). Simple main effect analysis of Target Position indicated significant differences among the 21 target positions in the -200 ms, *F*(20, 420) = 12.801, *p* < .001; 0 ms, *F*(20, 420) = 1.811, *p* = .018; and +200 ms, *F*(20, 420) = 4.219, *p* < .001, SOA conditions. Apparently, a small repulsion effect around the center of the screen was present in the 0 ms condition (see Figure 2). This might be due to the stronger salience of the cues over the target bars, because the cues were larger and appeared brighter than the targets. As a consequence, the observers’ attention was first directed to the cues and then to the targets; this “shift of attention” created the small repulsion effect observed in the data.

**Figure 3.**
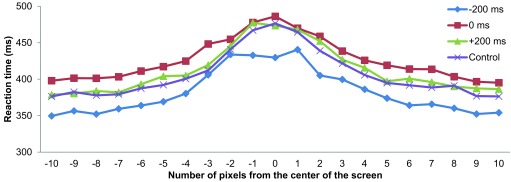
Average reaction times plotted against target positions in Experiment 1.

The judgment task for relative position was maximally difficult when the vertical bars appeared to be aligned with each other. This was reflected by longer RTs around the 0-pixel displacement condition (cf. Figure 3). A 4 × 21 (SOA × Target Position) repeated-measures ANOVA indicated significant main effects of SOA, *F*(3, 21) = 21.485, *p* < .001, and Target Position, *F*(20, 140) = 15.420, *p* < .001. The SOA × Target Position interaction was also significant, *F*(60, 420) = 1.666, *p* = .002. Figure 3 illustrates that in all four cue conditions, RT peaked when the bars appeared near the center position of the screen. Post-hoc comparisons using Ryan’s method revealed that the -200 ms condition had significantly shorter RT than the 0 ms, +200 ms, and control conditions (all at *p* < .05). In addition, participants had significantly longer RTs in the 0 ms condition than in the +200 ms and control conditions (both at *p* < .05). There was no significant difference in RT between the +200 ms and control conditions. An important result is that the RT in the +200 ms condition was shorter than that in the 0 ms condition. Figure 3 illustrates that the 0 ms, +200 ms, and control conditions showed similar and overlapping RTs, particularly in the seven central target positions.

**Figure 4.**
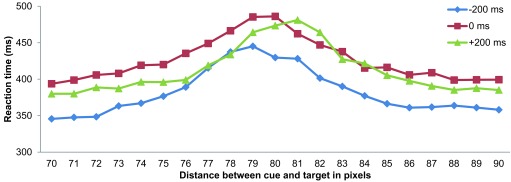
Average reaction times plotted against distance between cue and target in Experiment 1.

Figure 4 offers another perspective on the relationship between spatial distortion and RT. While Figure 3 shows RT collapsed across cue positions, Figure 4 shows the RT relative to cue location. For instance, in Figure 3, the position of “-10 pixels” considers the average of the RTs in the L cue condition (in which the top-left circle appeared 70 pixels to the left of the top bar, and the bottom-right circle appeared 70 pixels to the right of the bottom bar) and in the R cue condition (in which the top-right circle appeared 90 pixels to the right of the top bar, and the bottom-left circle appeared 90 pixels to the left of the bottom bar); note that in both cases, the target was in the -10 pixels configuration. However, in Figure 4, the position of “70 pixels” considers the average of the RTs in the L cue condition with the target appearing in the -10 pixels configuration (so that the top-left circle appeared 70 pixels to the left of the top bar, and the bottom-right circle appeared 70 pixels to the right of the bottom bar) and that of the R cue condition with the target appearing in the +10 pixels configuration (so that the top-right circle appeared 70 pixels to the right of the top bar and the bottom-left circle appeared 70 pixels to the left of the bottom bar). A plot of the average RT against the distance between the position cue and the target revealed that RT peaked at a position closer to the cue than the center in the -200 ms SOA condition (i.e., under the attentional repulsion effect) and further away from the cue than the center in the +200 ms SOA condition (i.e., under the attentional attraction effect). Considering the repulsion and attraction effects respectively present in these two conditions, the positions of the RT peaks seem to correspond to the locations where the target bars were perceived as aligned and therefore where the longest RTs were required for the observer to make the judgment.

A Gaussian function was fitted to the data shown in Figure 4 in order to determine the estimated distance between cue and target where the peak RT value occurred in the -200 ms, 0 ms, and +200 ms conditions for each observer using the nonlinear least squares function provided in R (version 2.15.1; R Development Core Team, 2012). A one-way ANOVA revealed a statistically significant difference between the means of the estimated positions of the RT peaks in the three SOA conditions, *F*(2, 14) = 9.065, *p* = .003. Post-hoc analysis adjusted for multiple comparisons using Ryan’s method identified significant differences in the estimated peak position between the -200 ms (*M* = 79.203, *SD* = 0.814) and +200 ms (*M* = 80.322, *SD* = 0.610) conditions, and between the 0 ms (*M* = 79.217, *SD* = 0.564) and +200 ms conditions (both at *p* < .05); however, no significant difference was found between the -200 ms and 0 ms SOA conditions.

## Experiment 2: Attentional capture controlled by color relevance of the cue to the target

In Experiment 2, we manipulated the strength of cue-induced attentional capture by presenting cues with colors matching or different from that of the targets. This manipulation allowed us to examine how the strength of attentional capture contributes to changes in the repulsion and attraction effects.

### Methods

#### Observers

Nine new naďve observers plus one of the authors (R.A.) participated in this experiment. All had normal or corrected-to-normal vision and provided informed consent prior to the experiment.

#### Apparatus, stimuli, and experimental procedures

The experimental apparatus, stimuli, and procedures were basically identical to those used in Experiment 1. Experiment 1 revealed that trials with target positions at the periphery of the screen did not generate significant repulsion or attraction effects; thus, such trials did not provide interesting data for interpretation. Therefore, to simplify the procedures, we only included the central seven target positions in Experiment 2. In addition to this change, in Experiment 2, the color of the target was always red, while the color of the cue could be either red or green, presented in a random order (the luminances of both the red and green cues were controlled at 3.00 cd/m^2^ in order prevent different levels of attentional capture from being caused by differences in luminance). The task of the observer remained the same: to judge whether the upper target bar appeared to be to the left or right of the bottom bar by pressing either the left or right mouse button as quickly as possible when the target disappeared. As in Experiment 1, RT data were collected for each trial. Each observer completed 224 trials (4 SOA conditions × 2 cueing conditions × 2 cueing colors × 7 target positions × 2 repetitions; in pseudorandom order) in each block. There were 10 blocks in total (total number of trials = 224 trials/block × 10 blocks = 2,240 trials), and each block took about 10 min to complete. Breaks of at least 5 min were given between each block.

### Results

**Figure 5.**
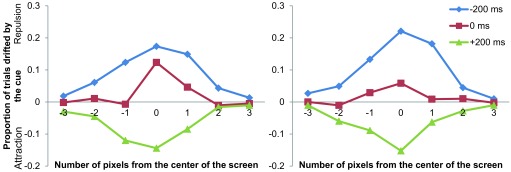
Magnitudes of attentional repulsion and attraction effects plotted against target positions for trials with cues colored the same as (left panel) and different from (right panel) the target in Experiment 2.

The attentional repulsion and attraction effects were computed in the same way as in Experiment 1, and the data from trials with RT shorter than 200 ms or longer than 1,000 ms were excluded from analysis (ave-rage percentages of trials discarded from each of the conditions were as follows: same color trials: -200 ms = 3.10%, 0 ms = 2.18%, +200 ms = 2.78%, control = 2.30%, overall = 2.59%; different color trials: -200 ms = 2.82%, 0 ms = 2.10%, +200 ms = 2.66%, control = 2.46%, overall = 2.51%). The data from one naďve observer was excluded because we found that the percentage of discarded trials was high as 18% on average for both the same color and different color trials. (We found that most of the discarded trials were rejected because the RTs were longer than 1,000 ms; and we believed that the observer must rely on short-term memory instead of instant perception in order to make the response in these trials with the extraordinarily long RTs. As our aim was to investigate the perception in the attentional repulsion and attraction effects, we wanted to minimize the effects from other factors such as memory; so we excluded her data from the analyses.) The magnitudes of the attentional repulsion and attraction effects are shown separately for the trials with red and green cue colors (the same as and different from the color of the target, respectively) in Figure 5.

A 2 × 3 × 7 (Cue Color × SOA × Target Position) repeated-measures ANOVA was conducted to examine the magnitudes of the attentional repulsion and attraction effects obtained in Experiment 2. The main effects of SOA, *F*(2, 16) = 24.544, *p* < .001, and Target Position, *F*(6, 48) = 3.297, *p* = .009, and their interaction, *F*(12, 96) = 11.390, *p* < .001, were statistically significant. In addition, the Cue Color × SOA interaction, *F*(2, 16) = 5.932, *p* = .012, and the Cue Color × SOA × Target Position interaction, *F*(12, 96) = 2.573, *p* = .005, were also significant. Post-hoc analysis adjusted for multiple comparisons using Ryan’s method confirmed the significant differences in effect magnitudes between the -200 ms and 0 ms conditions, between the -200 ms and +200 ms conditions, and between the 0 ms and +200 ms conditions (all at *p* < .01). Examining the significant Cue Color × SOA interaction, simple main effect analysis on SOA showed significant differences among the two cue colors in the -200 ms condition, *F*(1, 24) = 4.285, *p* = .049, while no significant differences were detected in the 0 ms, *F*(1, 24) = 1.918,*p* = .179, or +200 ms, *F*(1, 24) = 0.029, *p* = .867, conditions.

**Figure 6.**
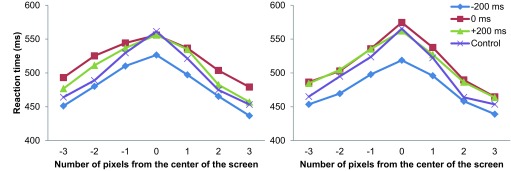
Average reaction times plotted against target positions for trials with cues colored the same as (left panel) and different from (right panel) the target in Experiment 2.

For RT data, Figure 6 indicates that the 0-pixel displacement position showed the longest RT among all target positions for both the same color and different color conditions. A 2 × 4 × 7 (Cue Color × SOA × Target Position) repeated-measures ANOVA showed significant main effects of SOA, *F*(3, 24) = 12.928, *p* < .001, and Target Position, *F*(6, 48) = 23.290, *p* < .001. The main effect of cue color and all other interactions were not significant, suggesting that cue color did not remarkably influence RT in various conditions. Post-hoc analysis conducted using Ryan’s method reported significantly shorter RTs in the -200 ms condition than in any other condition (all at *p* < .01); furthermore, the 0 ms condition showed significantly longer RTs than the control condition (*p* = .011).

**Figure 7.**
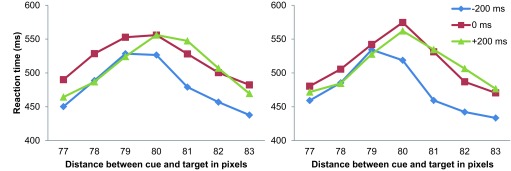
Average reaction times plotted against distances between cue and target for trials with cues colored the same as (left panel) and different from (right panel) the target in Experiment 2.

Figure 7 plots the average RT against the distance between the position cue and target separately for the same color and different color cue conditions. Similar to the results of Experiment 1, the RT peak shifted according to the SOA condition: A negative SOA value shifted the RT peak to a position closer to the cue, whereas a positive SOA value shifted the peak to a position further away from the cue.

A Gaussian function was fitted to the data using the nonlinear least squares function provided in R (version 2.15.1; R Development Core Team, 2012) to determine the estimated distance between cue and target where the peak RT value was at, in the -200 ms, 0 ms, and +200 ms conditions for each observer. A 2 × 3 (Cue Color × SOA) repeated-measures ANOVA revealed a significant main effect of SOA, *F*(2, 16) = 13.946, *p* < .001, and a significant Cue Color × SOA interaction, *F*(2, 16) = 8.229, *p* = .004, but the main effect of cue color was not significant, *F*(1, 8) = 0.795, *p* = .399. Post-hoc analysis performed using Ryan’s method reported significant differences in the estimated positions of the RT peak between the -200 ms (*M* = 79.260, *SD* = 0.527)and 0 ms (*M* = 79.808, *SD* = 0.358) SOA conditions, between the -200 ms and +200 ms (*M* = 80.304, *SD* = 0.237) SOA conditions, and between the 0 ms and +200 ms SOA conditions (all at *p* < .03). For the Color × SOA interaction, simple main effect analysis on SOA revealed significant differences in the estimated peak RT position among the three SOA conditions for both the same color and different color conditions. Post-hoc analysis using Ryan’s method reported significant differences between the -200 ms (*M* = 79.418, *SD* = 0.591) and +200 ms (*M* = 80.326, *SD* = 0.243) conditions, and between the 0 ms (*M* = 79.719,*SD* = 0.464) and +200 ms conditions in the same color condition. For the different color condition, significant differences were found between the -200 ms (*M* = 79.103, *SD* = 0.511) and 0 ms (*M* = 79.897, *SD* = 0.320) conditions, and between the -200 ms and +200 ms (*M* = 80.282, *SD* = 0.257) conditions (all at *p* < .01).

## Discussion

The present results have several important implications for the temporal characteristics of processing in the attentional repulsion and attraction effects. One highlight of the results is that the average RTs in the +200 ms SOA condition were comparable to (and generally shorter than) those in the 0 ms SOA condition in Experiment 1 (cf. Figure 3). This result supports the notion that delayed cue presentation in the +200 ms condition did not lead to delayed processing and thus delayed response. It is also worth noting that the location of the RT peak was shifted toward and away from that of the cue when attentional repulsion and attraction were expected, respectively (cf. Figure 4); this indicates that RTs depended on perceived relative positions rather than on physical positions. One straightforward interpretation of the present results is that the rate of attentional processing of the cue was faster than the localization process of the target. The localization process of the target might take a sufficiently long time (at least more than 200 ms) to an extent that the attentional influence from the cue (which we believe to occur at an unconscious level; Au, Ono, & Watanabe, 2013) could reach and be inserted into the ongoing process of target localization before it finishes. As shifts of visual attention can occur with very short latencies (less than 200 ms; Mackeben & Nakayama, 1993), it is plausible that dynamic shifts of visual attention may influence the localization pro-cess before the conscious representation of the visual targets is formed, resulting in the comparable RT values observed among the +200 ms, 0 ms, and control conditions. The present data fit well with the prediction made with the two-process assumption in conjunction with the dynamics of attentional shifting mentioned in the Introduction section (Müsseler & Aschersleben, 1998; Ono & Watanabe, 2011), supporting the hypothesis that processing of the attentional shift between cue and target operates at an unconscious level (Au et al., 2013), while the observer is only conscious of the final, settled state of configuration after the attentional shift has been totally completed.

In addition to dynamic attentional shifting, other accounts, such as the prior entry hypothesis (Schneider & Bavelier, 2003; Shore, Spence, & Klein, 2001) which suggests that attention speeds up sensory processing so that attended stimuli are perceived (i.e., come to consciousness) more quickly than unattended ones could possibly explain the observed results. The prior entry hypothesis could explain the repulsion effect by suggesting that attentional capture by the cue speeds sensory processing of the cued side more than that of the uncued side. This prior entry advantage for the cued side might induce propagation of visual signals towards the uncued side, such that the subsequently presented target might be “repelled” along this direction of propagation, leading to an apparent repulsion effect (the attraction effect could be explained in a similar way). The prior entry hypothesis offers an explanation similar to dynamic attentional shifting, which has been advocated by Ono and Watanabe (2011) and others. While both of these hypotheses might explain the observed data, future investigation is required to determine whether they represent the true underlying mechanisms of the attentional repulsion and attraction effects.

With reference to the present results suggesting that the dynamic attentional shift between the cue and the target is mediated by unconscious processes, a number of past studies have discussed the possibility that attention operates at unconscious levels and influences subsequent target localization. One illustrating line of such research was conducted by Scharlau and colleagues. Using unmasked and masked stimuli, Scharlau (2002) found that leading (but not trailing) primes influenced temporal order perception, supporting the notion that the phenomenon of perceptual latency priming is driven by attentional mechanisms. Experiments elucidating the Fehrer-Raab effect suggested that non-conscious stimuli might be processed in a way that allows the observer to respond without or in advance of the establishment of conscious perception of the scene (Neumann & Scharlau, 2007). This point is relevant to the two-process assumption of visual processing advocated by Müsseler and Aschersleben (1998) and explains the present results that delayed presentation of the cue did not delay responses. In a recent study using rapid visual serial presentation (RSVP), Hilkenmeier, Olivers, and Scharlau (2012) demonstrated that cueing attention toward either of two serially presented targets strongly affected order errors, providing new support to the prior entry hypo-thesis in explaining illusions related to temporal attention, in addition to the well-established explanation for illusions related to spatial attention. In another recent study, Priess, Scharlau, Becker, and Ansorge (2012) employed the flash-lag effect to demonstrate that sequential coding of two stimuli could lead to mislocalization the direction of which could be predicted from the coding of the order of the moving object relative to the flash. Their proposed attentional sequential-coding explanation was apparently derived from the prior entry hypothesis. These studies offered the perspective to explain the attentional repulsion and attraction effects concerned in the present study by suggesting that, due to attentional modulation, the information at different spatial locations is coded by the visual system at different moments; while this does not affect the overall conscious perception of the observer, attentional modulation is processed at an unconscious level.

Studies on top-down attentional control have also provided converging lines of evidence to support the idea that attention can operate at unconscious levels. Previous research has suggested that in a spatial cueing paradigm, attentional capture is contingent on attentional control settings induced by task demands. In other words, if the color of a task-irrelevant cue matches the top-down search settings for the target, attention is captured towards the cue to a greater extent than when the color of the cue does not match the top-down search settings (Folk, Remington, & Johnston, 1992). In the study on the attentional attraction effect, Ono and Watanabe (2011, Experiment 2) presented colored cues (red and green) that occupied both possible directions of target position shift and instructed observers to attend to cues in of one of the colors. By engaging attention with the endogenous intention of observers in such a top-down manner, the experiment showed that the positional distortion of the target depended on where attention was focused, supporting the idea that the attentional distortion effects were driven by attentional processes. Such goal-driven attentional capture induced by color can occur even when the color is rendered invisible to the observer by masking (Ansorge, Horstmann, & Worschech, 2010; Ansorge, Kiss, & Eimer, 2009); further, stimuli at the non-conscious level are processed at an individual-feature level, while stimuli that have reached the conscious level could be additionally processed at the whole-object level (Tapia, Breitmeyer, & Shooner, 2010). This evidence is in line with the previous proposal that attention can operate at the unconscious level (i.e., that attention and awareness can be dissociated; Koch & Tsuchiya, 2007; Lamme, 2003; Woodman & Luck, 2003).

The present results resemble those of an RT study of the apparent motion phenomenon by Cowan and Greenspahn (1995). In that study, the RT of reporting the middle point of the journey of apparent motion did not differ from that of reporting the ending position. This means that the consciously perceived sequence of events does not necessarily correspond to the temporal sequence of information processing in the brain and the behavioral consequences. Experiments on temporal binding have also demonstrated that the perceived asynchrony of visual events might not reflect either neural processing or the real-time sequence of physical stimulus presentation (Moutoussis & Zeki, 1997; Nishida & Johnston, 2002). In this regard, the present results (that delayed cue presentation in the +200 ms SOA condition did not delay responses compared with simultaneous presentation) are in line with these previous studies. However, it is important to note that the attentional repulsion and attraction effects are distinct phenomena from apparent motion, although they are similar in terms of stimulus configuration (i.e., both phenomena involve brief stimulus presentations at two separate positions and instances). In the attentional repulsion and attraction effects, observers were asked only to judge the position of the target stimulus, and regard the cue as irrelevant to the task. Under these conditions, the effects can still occur even the target and cue stimuli are largely different in their shape or salience (and therefore do not favor the occurrence of apparent motion). Supporting this distinction, in the original demonstration of the attentional repulsion effect, motion distractors (which had an identical appearance to the attentional cue) that induced apparent motion in the opposite direction from the repulsion effect did not produce a noticeable reduction in the repulsion effect (Suzuki & Cavanagh, 1997, Experiment 3). In addition, accumulated evidence shows that the repulsion and attraction effects are caused by attentional shifts. For example, attentional manipulations that produce known effects on RT can also produce analogous spatial repulsion (Pratt & Arnott, 2008).

Experiment 2 of the present study with colored cues also provided strong support against the idea which posits that the repulsion and attraction effects are due to perceptual apparent motion rather than attentional processes. The results of Experiment 2 suggest that the different color condition created stronger spatial distortion effects than the same color condition. One possible reason for these results is that, since the target judgment task employed in the present study is not a search task that demands attention in order to find a designated stimulus, the use of a differently colored cue might elicit a stronger attentional capture in a bottom-up manner than the same color condition. Since color is processed in the preattentive stage of early visual processing (Treisman, 1985), it serves to guide the later deployment of limited attentional resources (Wolfe, 1994). Similar to the “pop-out effect”, in which a unique target among a set of homogenous objects in a visual search task (e.g., a target with a different color from the rest of the display) can be detected rapidly (Treisman, 1985; Wolfe, 1994), the different color cue in our experiment “pops out” from the display and might attract the observer’s attention more strongly than the same color cue. Our results support the notion that the attentional repulsion and attraction effects are not driven by principles of apparent motion, but are attentional in nature. If apparent motion actually determined the attentional repulsion and attraction effects, stronger effect magnitudes would have been expected in the same color than the different color condition, as two objects of the same color should have created a more vivid perception of apparent motion than two differently colored objects (color correspondence; Green, 1989). On the other hand, one may also interpret the stronger repulsion effect observed in the different color trials of the -200 ms SOA condition in terms of the notion of attentional deallocation proposed by Theeuwes (1994). That framework posits that attentional processes occur in two stages: A salient stimulus (the colored cue) first captures attention in a bottom-up manner regardless of whether the color matches the top-down controlled set of target features, followed by the process of deallocating attention from the task-irrelevant cue in order to prepare for a fast response to the target. Since it is easier to discriminate a cue with a different color from the target than one with the same color as the target, the deallocation process could therefore occur at an earlier moment in the different color condition than in the same color condition. The more efficient attentional deallocation from the cue in the different color than the same color condition might have facilitated the repulsion effect observed in the different color condition. This “advantage” of earlier deallocation from the cue disappeared in the +200 ms SOA condition because the cue was presented after the target, resulting in no observable difference in the magnitude of the attraction effect.

The present study examined the relationship between attentional effects and RT functions; this has also been the subject of several previously published studies. For example, in the study by Shore et al. (2001), the attention of observers was exogenously or endogenously oriented to the left, right, or center of the screen, and observers reported the order of two serially presented horizontal and vertical line segments (on the same or different sides). Analyses of RT functions revealed that the point of maximum RT generally shifted in the direction that would be expected if attentional capture accelerated perceptual arrival times, matching well with the data derived from an analysis of the point of subjective simultaneity (PSS) and thus providing support for the prior entry mechanism. While Shore and colleagues (2001) suggested that the peak of RT functions could mark the PSS in temporal order judgment tasks, Scharlau (2007) remarked that the peak point of these functions is difficult to statistically evaluate since judgment times are often highly variable; further, when the RT function has a shallow slope, it can be difficult to locate the peak position. As argued by Scharlau (2007), the peak of an RT function might just represent the point of maximal uncertainty instead. Nevertheless, analysis of RT distributions using novel methods may provide new insights for deriving models of attentional processes. One such example is from Hübner, Steinhauser, and Lehle (2010), who analyzed the cumulative RT distributions of correct and incorrect responses in three flanker task experiments, and derived the dual-stage two-phase model of selective attention which differentiates the initial filtering of information with limited selectivity at the early stage from the more efficient, category-based filtering that takes place at a later stage. Future studies could examine, for example, how the visual similarity between the cue and the target across different dimensions might modulate attentional deployment, as reflected in RT distributions.

As a secondary finding, in the present study, RTs were significantly faster in the -200 ms SOA condition than in any of the other conditions (cf. Figure 3). This might be explained by the different nature of target presentation timing between conditions. In the -200 ms SOA condition, the target was always presented after a constant delay of 200 ms after the presentation of the cue. In every trial, cue presentation reliably notified the observer that the target would arrive shortly after a constant time interval. In a sense, compared with the 0 ms, +200 ms, and control conditions, observers were more ready for rapid response at the time of cue presentation in the -200 ms SOA condition. This might lead to the evenly faster RTs observed over all target positions in the -200 ms SOA condition than the other three conditions.

In conclusion, the present study has demonstrated that a physical delay in the timing of stimulus presentation does not necessarily lead to the same delay in response under the attentional attraction effect. The processing rate of the cue, presumably determined by a rapid shift of visual attention, might be more rapid than the target localization process and might have been inserted into the default process without affecting the overall response time. In addition, the present experiment showed that the retrospective alternation of positional representations (under +200 ms SOA) had effects similar to those of unaffected representations (with 0 ms SOA) on localization RT; namely, perceptually aligned lines were more difficult to localize than others (cf. Figure 4), indicating that spatial distortion caused by attentional attraction occurs at the perceptual rather than decisional level. Further investigation is necessary to clarify in detail the mechanisms underlying the perceptual phenomena involving this seemingly retrospective form of processing. One issue is the relationship between processing rate and stimulus saliency. We propose that attentional processing of the cue can be much faster than the joint perceptual processing of the cue and target because of the larger salience of the cue than the target in the present study. In future studies, the relative contributions of stimulus saliency and attentional processes (both of which are known to influence perceptual and behavioral latencies) to spatial distortion effects should be examined. Also, magnetoencephalography (MEG), having been applied in previous experiments to study the neural dynamics of visual perception, might be a suitable technique to closely examine the relationship between the physical onset of the stimulus, internal neural responses, and behavioral RT of the phenomenon concerned (Amano et al., 2006).
